# Routine Habitat Change: A Source of Unrecognized Transient Alteration of Intestinal Microbiota in Laboratory Mice

**DOI:** 10.1371/journal.pone.0047416

**Published:** 2012-10-17

**Authors:** Betty W. Ma, Nicholas A. Bokulich, Patricia A. Castillo, Anchasa Kananurak, Mark A. Underwood, David A. Mills, Charles L. Bevins

**Affiliations:** 1 Center for Laboratory Animal Science, School of Veterinary Medicine - Residency Program in Laboratory Animal Medicine, University of California Davis, Davis, California, United States of America; 2 Department of Microbiology and Immunology, School of Medicine, University of California Davis, Davis, California, United States of America; 3 Department of Food Science and Technology, University of California Davis, Davis, California, United States of America; 4 Department of Viticulture and Enology, University of California Davis, Davis, California, United States of America; 5 Department of Pediatrics, School of Medicine, University of California Davis, Sacramento, California, United States of America; Wadsworth Center, New York State Dept. Health, United States of America

## Abstract

The mammalian intestine harbors a vast, complex and dynamic microbial population, which has profound effects on host nutrition, intestinal function and immune response, as well as influence on physiology outside of the alimentary tract. Imbalance in the composition of the dense colonizing bacterial population can increase susceptibility to various acute and chronic diseases. Valuable insights on the association of the microbiota with disease critically depend on investigation of mouse models. Like in humans, the microbial community in the mouse intestine is relatively stable and resilient, yet can be influenced by environmental factors. An often-overlooked variable in research is basic animal husbandry, which can potentially alter mouse physiology and experimental outcomes. This study examined the effects of common husbandry practices, including food and bedding alterations, as well as facility and cage changes, on the gut microbiota over a short time course of five days using three culture-independent techniques, quantitative PCR, terminal restriction fragment length polymorphism (TRFLP) and next generation sequencing (NGS). This study detected a substantial transient alteration in microbiota after the common practice of a short cross-campus facility transfer, but found no comparable alterations in microbiota within 5 days of switches in common laboratory food or bedding, or following an isolated cage change in mice acclimated to their housing facility. Our results highlight the importance of an acclimation period following even simple transfer of mice between campus facilities, and highlights that occult changes in microbiota should be considered when imposing husbandry variables on laboratory animals.

## Introduction

A complex non-random community of colonizing microbes, termed microbiota, inhabits skin and mucosal surfaces [1]. Humans and other animals have coevolved with these microbes so as to not only tolerate, but also to require their presence for health and normal physiology [2]–[4]. In turn, the host provides the microbes a beneficial environment, creating a mutualistic relationship [2]. In the last decade, a combination of culture-independent experimental approaches and rapid advances in DNA sequence technologies have contributed to a wealth of knowledge on the composition and function of the microbiota [5], [6].

Microbial abundance and diversity are particularly striking in the mammalian intestine. While four kingdoms are represented in this community of colonizing microbes, the most abundant, and chief focus of investigations to date, are the bacteria [7]–[11]. The mammalian intestine harbors a complex bacterial community of at least 1,000 species, comprised mostly of members of the phyla *Bacteroidetes* and *Firmicutes*. The *Bacteroidetes* are Gram-negative bacteria, including numerous *Bacteroides*, *Cytophaga*, and *Flavobacterium* species, while the intestinal *Firmicutes* are Gram-positive bacteria, such as members of the *Enterococcaceae* and *Lactobacillaceae* families and the *Clostridia* class. Together, these taxa account for ninety percent or more of the nearly hundred-trillion bacteria in the gut. By harboring a vast, diverse, and dynamic bacterial population in the gut, the host acquires access to an enormous collection of microbial genes (a metagenome), encoding proteins and enzymes that aid in digestion, produce essential vitamins and nutrients, enhance intestinal development, and prime the intestinal immune system [3], [12]. The dynamic and intimate interactions between the host and its intestinal microbiota can have effects beyond the intestine [6], [12]–[15]. The microbiota can even affect neurobiological and behavioral phenotypes [16]. Some have used the term “the forgotten organ” to describe the gut microbiome, because it functions in such diverse physiological processes [15].

Dysbiosis, the alteration or imbalance in the microbiota, can create a niche for proliferation of virulent organisms to disrupt homeostasis and cause disease. However, even in the absence of overt pathogens, an imbalance can be detrimental to the host [13], [17]. A variety of environmental influences, as well as genetic host factors can cause dysbiosis, dramatically increasing susceptibility to acute infectious diseases [18] and to chronic inflammatory diseases [19], including obesity, diabetes, gastrointestinal cancers, atherosclerosis, inflammatory bowel disease, and asthma [20]–[30].

Mice are the most widely used species to model acute and chronic disease [31]. Many valuable insights on the association of the microbiota with disease stem from, and depend on, investigation of mouse models. As with humans, the gut microbial community in mice is relatively stable and resilient. Nevertheless, environment can influence the microbiota. Basic animal husbandry is an often-overlooked variable in research that can potentially alter experimental outcomes [32], [33]. Despite a wide appreciation that husbandry practices influence the microbiota [34], the specific contributions of routine practices are not well understood. This study examined the effects of food and bedding alterations, as well as the common husbandry practices of facility and cage changes, on the microbiota over a short time course of five days. Above the other variables, we report here that a simple intracampus facility and cage change has a significant, but previously unappreciated impact on the gut microbiota.

## Materials and Methods

### Mouse Husbandry and Experimental Design

All procedures were performed under a protocol approved by the UC Davis IACUC. Mice were housed according to the recommendations in the Guide for the Care and Use of Laboratory Animals of the National Institutes of Health. FVB mice, 7–10 weeks of age, were reared in a specific pathogen free (SPF) barrier facility and transferred to a conventional housing facility on day zero. Mice were maintained on a 12 hr light/12 hr dark cycle at 30–70% humidity and at 70–72°F ambient temperature. All animals were given free access to food and water, and were housed in same sex pairs in static micro-isolator cages. Water was purified using the Edstrom water acidification system (Edstrom Industries, Inc, Waterford, WI) with a pH in the range of 2–3, checked at time of bottle filling with monthly quality control testing for pH accuracy and bacterial growth. All cages and bedding were autoclaved according to institutional guidelines prior to animals being placed into them. Pathogen monitoring in the conventional housing facility utilized a sentinel program with quarterly screens to ensure the health of the animals. All testing of rodent sentinels in the housing rooms were negative through out the duration of the study. To assure strict consistency, one investigator (BWM) assumed all care, handling and maintenance of mice during the experimental protocol. Cage changes were made as indicated in the specific experiments in adherence to institutional guidelines and to mimic normal husbandry procedures.

**Figure 1:**
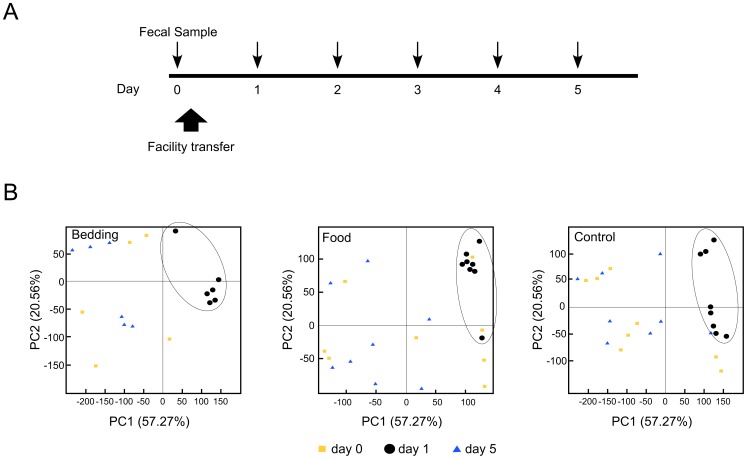
Analysis of stool microbiota changes following husbandry changes. A) Timeline of experimental approach. Fresh fecal samples were collected daily at each time point from individual mice (downward arrows). Day 0 denotes baseline samples obtained just prior to transfer from an SPF facility to a conventional housing facility (upward block arrow). Upon transfer, either bedding (group 1) or food (group 2) was changed (from corncob to Carefresh bedding, and Teklad 2918 to LabDiet 5058, respectively). Control mice (group 3) were also transferred and placed in a fresh cage, but maintained on the same type of food and bedding (corncob and Teklad 2918). Day 1 is 24 hours after the combined facility transfer/cage change for all mice. B) Principal coordinate analysis of Eubacterial-TRFLP data. Each data point represents a single mouse specimen at days 0 (yellow squares), 1 (black circles) and 5 (blue triangles) from mice in three experimental groups: bedding change (n = 6), food change (n = 8) and control (n = 8). A PCoA was performed using all data, and then graphed for each of the three experimental groups separately.

The experimental groups of mice used in this study were: “group 1”) Bedding change: mice (n = 6, 2F/4M) were changed from corncob bedding (Harlan Laboratories, Indianapolis, IN) to Carefresh bedding (Carefresh, Canon City, CO); “group 2”) Food change: mice (n = 8, 4F/4M) were changed from Harlan Teklad 2918 (Harlan) to Purina Lab Diet 5058 (MI Nutrition International LLC (LabDiet), St. Louis, MO); “group 3”) Control: mice (n = 8, 4F/4M) were housed on corncob bedding and maintained on Harlan Teklad 2918 diet; “group 4”) High fat diet change: mice (n = 8, 4F/4M) were changed from Purina Lab Diet 5058 to a 20% high fat diet by Open Source Diets (Research Diets, New Brunswick, NJ). Fresh fecal samples were collected just prior to the facility/cage change (day 0), and then daily for 5 days thereafter (Fig. 1A). After the facility/cage change on day 0, no further change in bedding, food or cage was made for any of the mice, and fecal material and waste was allowed to accumulate for the remainder of the 5-day experiment. Thus, mice in Group 3 (controls) experienced a facility/cage change on day zero, placing them in a new cage with fresh bedding and food, but were maintained on otherwise identical corncob bedding and Harlan Teklad 2918 diet.

A second independent cohort of mice (“group 5”, n = 7, 3F/4M) were similarly transported from the same SPF facility as the first group, but were placed in a different conventional housing location. Like Group 3, this cohort of mice was also housed on corncob bedding and maintained on Harlan Teklad 2918 diet. They remained in the conventional housing facility for 37 days to assess the effects of cage changes in acclimated mice (without concomitant facility change). During this time, cages were changed (by BMW) on days 0, 7, 14, 21, and 28, as part of normal husbandry. Fresh fecal pellets were collected daily and TRFLP analysis was performed on samples collected on day 0 (just prior to the facility/cage change), day 1, day 5, days 7 and 21 (just before routine cage changes), days 8 and 22 (one day after routine cage changes), and on days 12 and 26 (five days after routine cage changes).

### Specimen Collection

Fecal pellets were freshly collected from each mouse daily in the early morning after timed lighting was turned on. Fecal pellets were promptly weighed and suspended in RNAlater (Ambion Life Technologies, San Diego, CA). The fecal samples in RNAlater were incubated overnight at room temperature and then stored at −80°C until processed for DNA extraction, as previously described [35].

### DNA Extraction

Fecal samples were thawed, and the pellets were washed once in 750 µl ice cold PBS, resuspended in 200 µl lysis buffer (20 mM Tris HCl (pH 8), 2 mM EDTA, 1.2% Triton X-100, 40 mg/ml lysozyme) and then transferred to lysis matrix tubes (MP Biomedical: Solon, OH, Lysing Matrix B, 0.1 mm silica spheres). The resulting suspension was incubated at 37°C for 30 min and then disrupted using a Mini-beadbeater-16 vortex (BioSpec Products, Bartlesville, OK) for 2 min according to the manufacturer’s protocol. After vortexing, DNA was extracted using the Qiagen DNA stool kit (Qiagen, USA) according to manufacturer’s protocol, including the modification for stool pathogen detection.

### Quantitative PCR (qPCR)

Oligonucleotide primer pairs designed to detect the 16S ribosomal DNA for bacterial groups within the *Firmicutes* and *Bacteroidetes* phyla were used as described previously [35]. Specifically, the assays for subphyla of *Firmicutes* included *Lactobacillus, Clostridium leptum* and *Eubacterium rectale*; the subphyla of *Bacteroidetes* were *Bacteroides* and *Mouse Intestinal Bacteroides.* The qPCR used fecal DNA (∼20–30 ng) in a 10 µl reaction containing 4 mM MgCl_2_, 0.5 µM of each primer and 1X LightCycler-Fast Start DNA Master SYBR Green I mix (Fast Start SYBR kit, Roche Diagnostics, Mannheim, Germany) and was performed using the Roche LightCycler 2.0 (Roche Diagnostics). Previously published primer pairs [35] were used with slight modification of the annealing temperatures shown in Table S1. Values for each assay were determined using standard curves constructed with reference bacterial DNA specific for each bacterial group as described [35].

### qPCR Data Analysis

Quantitative estimates for individual bacterial sample groups were normalized to the total abundance of bacteria estimated using the kingdom-specific *Eubacteria* assay. Data for each bacterial group were analyzed either from individual mice at two time points, or between experimental cohorts at a specific time point. For assessment of changes in individual mice over time, the normalized values were analyzed using a paired t-test. For comparison between groups, the averaged values were compared using a two-tailed Student’s t-test.

### Eubacterial Terminal Restriction Fragment Length Polymorphism (TRFLP)

Eubacterial-TRFLP analysis was performed following protocols previously described [36]. Briefly, a PCR reaction was performed using the purified fecal DNA (∼5–40 ng) and the Fast Start Taq PCR system (Roche) on a Veriti 96 well thermocycler (Applied Biosystems, Foster City, CA). The oligonucleotide primers, 1492R (5′-GGTTACCTTGTTACGACTT-3′) and Uni331F (5′-[FAM]-TCCTACGGGAGGCAGCAGT-3′) were used at a final concentration of 0.2 µM. The PCR conditions were: initial denaturation at 94°C for 5 min, followed by 35 cycles of 94°C for 30 sec, 50°C for 30 sec (annealing) and 72°C for 90 sec (extension). The appropriate size of the PCR product was verified using agarose gel electrophoresis (1% w/v in TAE buffer) using a 100 BP DNA ladder (Roche). The PCR product was purified using a Qiagen PCR purification kit according to the manufacturer’s protocol. Approximately 500 ng of the purified PCR DNA product was then used for each restriction enzyme digestion with either Alu I, Hae III, Hha I, or Msp I restriction enzymes (Promega, Madison, WI), according to the supplier’s recommended buffers and protocols. The reactions were terminated by incubation at 65°C for 20 minutes to inactivate the restriction enzymes. Each restriction digestion sample was analyzed for DNA fragment length and quantity using an ABI 3100 Capillary Electrophoresis Genetic Analyzer (Applied Biosystems, Carlsbad, CA) at the University of California, Davis Sequencing Core Facility.

### Lactic Acid Bacteria (LAB)-TRFLP

LAB-TRFLP was performed as previously described [37]. PCR was performed similarly to the Eubacterial-TRFLP. Oligonucleotide primers were 5′-[HEX] GGCGGCGTGCCTAATACATGCAAGT-3′ and 5′-TCGCTTTACGCCCAATAAATCCGGA-3′ with a final concentration of 0.2 µM. The PCR conditions were: initial denaturation at 94°C for 5 min, followed by 30 cycles of 94°C for 45 sec, 66°C for 30 sec (annealing) and 72°C for 45 sec (extension). Appropriate product size was verified using the same conditions as the above TRFLP protocol. Approximately, 150–500 ng of DNA was used for restriction digest with either Mse I or Hpy118 I. Fragment analysis was performed as previously stated at the University of California, Davis Sequencing Core Facility.

### Next Generation Sequencing (NGS) Library Construction

Library preparation and data analysis were performed as described previously [38]. Briefly, the V4 domain of bacterial 16S rDNA was amplified using primers F515 (5′-CACGGTCGKCGGCGCCATT-3′) and R806 (5′-GGACTACHVGGGTWTCTAAT-3′) [39], both modified to contain an Illumina adapter region, and, on the forward primer, an 8 bp barcode to enable sample multiplexing. The sequence of V4 primers and barcodes is presented in Table S2. The PCR reactions contained 5–100 ng DNA template, 1X GoTaq Green Master Mix (Promega, Madison, WI), 1 mM MgCl2, and 5 pmol of each primer. The reaction conditions consisted of an initial 94°C for 3 min followed by 35 cycles of 94°C for 45 sec, 50°C for 60 sec, and 72°C for 90 sec, and a final extension of 72°C for 10 min. All samples were amplified in triplicate and combined prior to purification. The PCR products were purified using the Qiaquick PCR purification kit (Qiagen, Valencia, CA), quantified using PicoGreen dsDNA reagent (Invitrogen, Grand Island, NY), mixed at equimolar concentrations, and gel purified using the Qiaquick gel extraction kit (Qiagen). The purified libraries were submitted to the UC Davis Genome Center DNA Technologies Core for cluster generation and 150 bp paired-end sequencing on the Illumina GAIIx. Image analysis, base calling, and error estimation were performed using CASAVA 1.8.

### TRFLP Data Analysis

Electropherogram traces were visualized using the program Peak Scanner v1.0 (Applied Biosystems, Carlsbad, CA) with a baseline detection value of 10 fluorescence units. True peaks were identified from noise by filtration and clustering using the scripts and analysis protocols designed by Abdo and colleagues [40] in R software. Operational taxonomic units were assigned based on an *in silico* digest database generated by the virtual digest tool from MiCA [41] of good-quality 16S rDNA gene sequences compiled by the Ribosomal Database Project Release 10 [42], [43], allowing up to 3 nucleotide mismatches within 15 BP of the 5′ terminus of the forward primer. Principal coordinates were computed and visualized from Euclidean distance of raw Msp I digest TRFLP data (prior to taxonomic classification/grouping) using QIIME [44]. For assessment of the abundance of *Lactobacillales* over time, the data were analyzed using repeated measures ANOVA.

### NGS Data Analysis

Raw Illumina fastq files were demultiplexed, quality-filtered, and analyzed using QIIME v1.5.0 [44]. The 150-bp reads were truncated at any site of more than three sequential bases receiving a quality score <1e−5, and any read containing ambiguous base calls or barcode/primer errors were discarded, as were truncated reads containing <75 consecutive high-quality base calls. Operational Taxonomic Units (OTUs) were assigned using UCLUST [45] with 97% pairwise identity. OTUs were classified taxonomically using a QIIME-based wrapper of the Ribosomal Database Project classifier [46] against the Greengenes 16S rDNA sequence database [47], using a 0.80 confidence threshold. OTUs comprising less than 0.001% of total sequences for each run were removed prior to further analysis.

## Results

To investigate how husbandry variables might influence microbiota, mice reared in a SPF barrier facility were transferred across campus to a conventional housing facility and assigned to group 1 (bedding change), group 2 (food change) or group 3 (control). At day 0, all mice were placed in a new cage to commence the experiment (Fig. 1A, see Materials and Methods for precise details). Fresh fecal samples were collected just prior to the facility/cage change (day 0), and then daily for 5 days thereafter (Fig. 1A). Bacterial DNA was extracted from the stool samples for Eubacterial-TRFLP and qPCR analysis. A principal coordinate analysis (PCoA) plot of the TRFLP data was generated to analyze changes in composition of the microbiota in mice from each experimental group at three time points, days 0, 1, and 5 (Fig. 1B). Each data point represents analysis of the microbiota of an individual mouse at a specific time point and provides an unbiased measure of variation of one sample compared to all others in this experiment. The PCoA plots show a striking alteration in the composition of microbiota at day 1, following the initial facility transfer and cage change. This alteration of the microbiota is evident as a shift from a diffuse scatter of data points at day 0 to a tightly clustered pattern at day 1 in all three groups. At day 5, the PCoA plots for all animals in each group showed a return to approximate the original diffuse pattern seen at day 0 (Fig. 1B). Contrary to our expectations, the PCoA indicated that the bedding and diet changes had a negligible effect on the microbiota. Rather, the analysis showed that mice in all three groups manifested a similar reshaping of their microbiota composition at day 1, indicating a significant influence of the habitat change on the microbiota.

**Figure 2:**
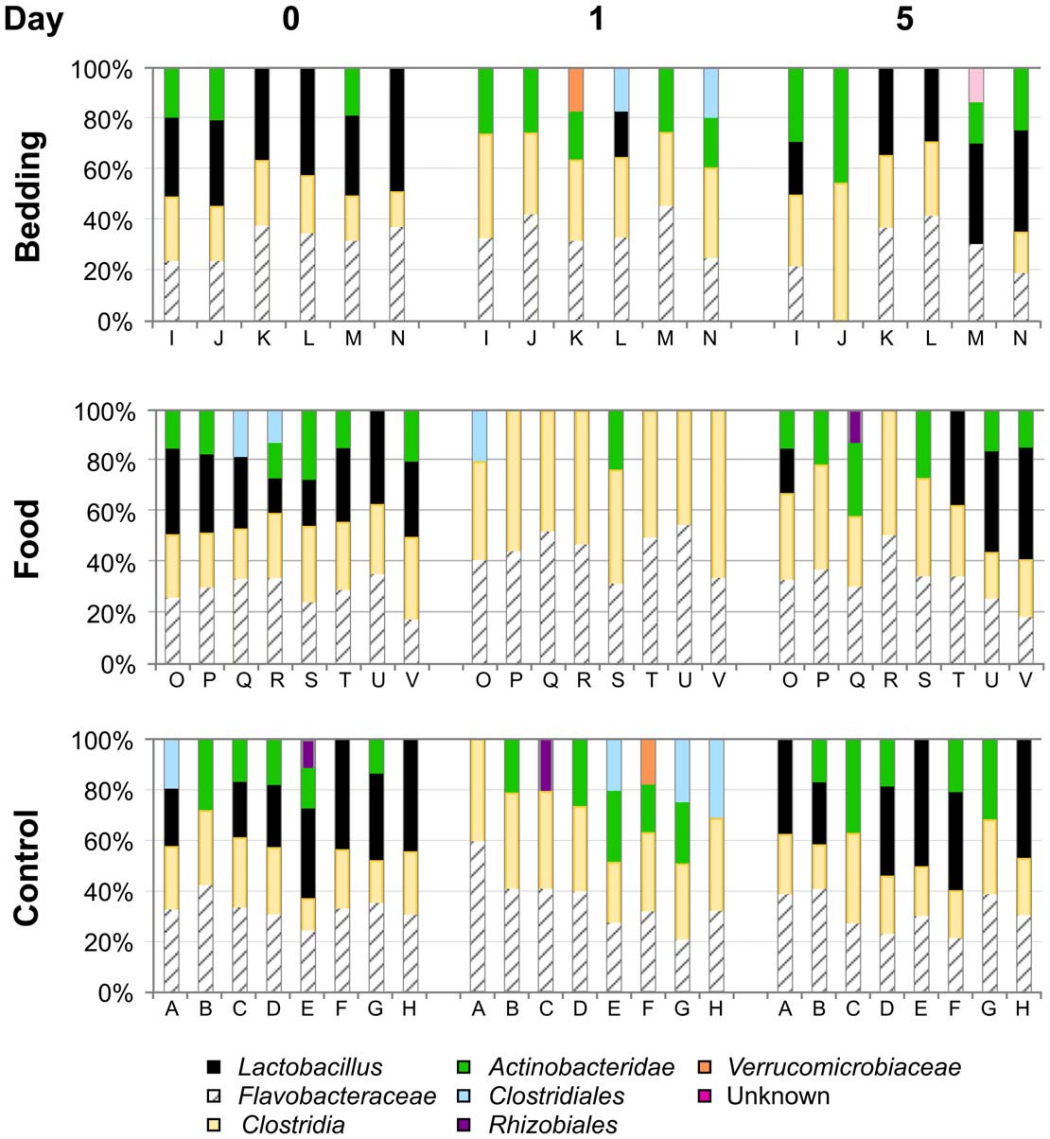
Taxonomic plot of bacterial populations derived from Eubacterial-TRFLP analysis for each mouse in groups 1–3. DNA isolated from stool of each mouse shown in Fig. 1 was analyzed by Eubacterial-TRFLP as described in the methods. Using these data, operational taxonomic units were assigned based on an *in silico* digest-database to estimate relative proportions of each major bacterial group present in the samples. Note that at day 1, there appears to be a loss of the Lactobacillus bacterial group (black) in 21/22 specimens. By day 5, there is a return to a profile of bacterial composition similar to that observed at day 0 (baseline) for all three groups.

A taxonomic plot of the TRFLP data assesses operational taxonomic units and proportions of these bacteria present in each sample. Analysis of individual taxonomic groups at day 1 shows an apparent disappearance of *Lactobacillales* in 21 of 22 mice with a concomitant expansion of the family *Flavobacteraceae* and class *Clostridia* (Fig. 2). By day 5 the microbial population diversifies to resemble the day 0 pattern, including a return of the *Lactobacillales* taxon in all three experimental groups (Fig. 2).

A similar pattern of change was also observed in an independent experiment with a separate cohort of seven mice, and while *Lactobacillales* decreased in abundance at day 1 in this experiment, their reduced numbers remained detectable (Fig. S1). The abundance of Lactobacilli in stool at days 0, 1 and 5 was compared using repeated measures ANOVA. For the 8 mice in group 3 (Fig. 2, mice A–H) the mean relative proportions of Lactobacilli at these time points were 28%, 0% and 29%, respectively (p<0.001), and for the 7 mice in the supplementary group (Fig. S1) these mean proportions were 15%, 7.4% and 17%, respectively (p<0.05). Thus, in both of these independent experiments we observed a significant transient decrease in Lactobacilli following the facility/cage cage.

To quantify select bacterial populations in each sample from groups 1–3, real-time qPCR of the microbiota was analyzed. Quantification of select bacterial populations, normalized to a universal bacterial assay, *Eubacterium*, substantiated that significant differences existed for individual mice between day 0 and day 1 (Fig. S2A). A comparison of the mice in groups 1–3 at day 5 did not detect any significant differences in bacterial populations attributable to either the food or bedding changes (Fig. S2B). The profile proportions were indistinguishable as compared to the control group (Fig. S2C), consistent with the PCoA and taxonomic analysis of TRFLP data (Figs. 1B, 2 and S1).

It was somewhat unexpected that we did not detect a statistically significant difference in microbiota when the mouse diet was changed from Harlan Teklad 2918 to Purina Lab Diet 5058 (group 2). Therefore, to address whether our approach could detect an altered microbiota at day 5 following a dietary change previously reported by other laboratories to alter microbiota [48]–[50], animals were switched from a standard maintenance rodent chow to a specially formulated high-fat diet, consisting of ∼20% crude fat (group 4). After 5 days, stool microbiota was analyzed by PCoA of TRFLP data. Control animals maintained on the standard diet (n = 7), displayed a similar diffuse pattern at both day 0 and 5 (Fig. S3A). In contrast, microbiota of mice switched to a high fat diet showed a dramatic shift along PC1 from the diffuse pattern at day 0 to a cluster at day 5 (Fig. S3B). This pattern is seen in mice whether they were on corncob or Carefresh bedding (Fig. S3B). Consistent with previously published reports [48]–[50], quantitative PCR analysis demonstrated a decrease in *Bacteroidetes* and an increase in *Firmicutes* in the mice on a high fat diet (Fig. S4).

**Figure 3:**
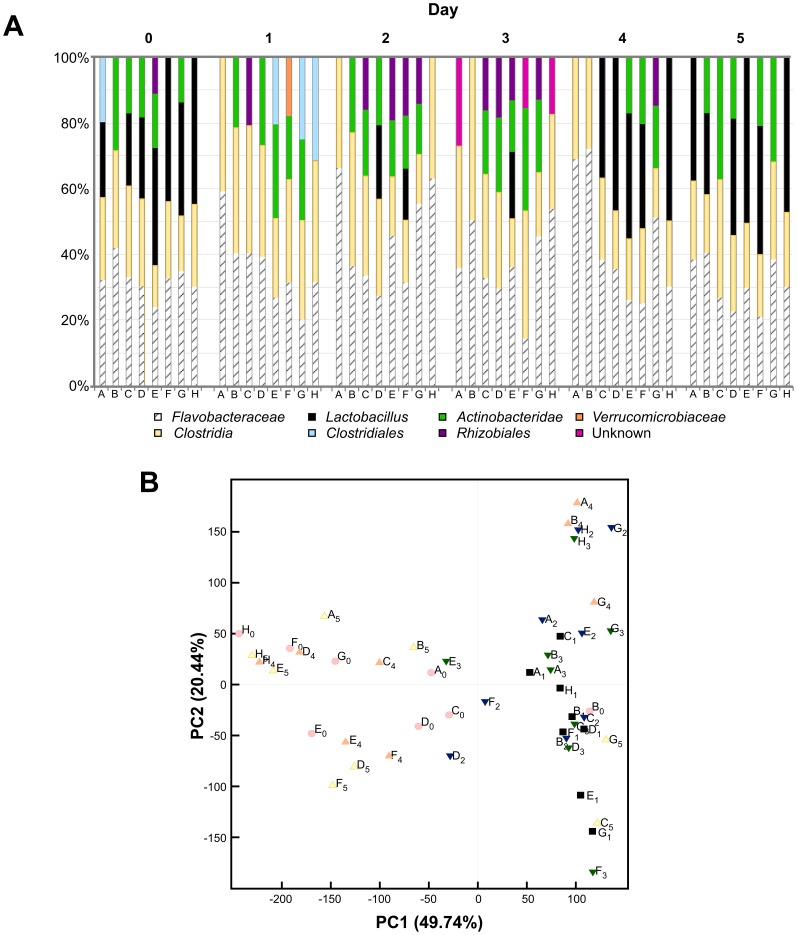
Eubacterial-TRFLP analysis of fecal microbiota in mice following a combined facility/cage change. A) Taxonomic plot of bacterial profiles daily from day 0 to day 5. A facility/cage change occurred on day 0 after stool was sampled (see Fig. 1A for timeline). DNA isolated from the feces of each mouse in the control group (group 3, n = 8) was analyzed by TRFLP as described in the methods. Mice A, B, G, H were males; C, D, E, F were females. Note that over time, the group of *Lactobacillus* initially lost at day 1, returns to the population profile in 5 of 8 mice by day 4, and 7 of 8 mice by day 5. B) Principal coordinate analysis of TRFLP data for control mice over time. Each data point represents a single mouse specimen at days 0 (pink circles), 1 (black squares), 2 (blue inverted triangles), 3 (green inverted triangles), 4 (orange triangles) and 5 (yellow triangles) from mice in the control group analyzed in A. Note, the shift and clustering of data at day 1, gradually returns to resemble the original pattern by days 4 and 5.

To further interrogate the significant microbiota changes following the facility/cage change in Fig. 1A, we performed TRFLP analysis of stool samples from the control group (group 3) at days 2, 3 and 4. The dramatic loss of microbial diversity detected at day 1 was followed by a gradual return to resemble baseline by day 4, with *Lactobacillus* detected in most individuals at this time point (Fig. 3A). By day 5, the microbiota returned to the pattern observed at day 0 (Fig. 3B). This pattern is also apparent when these data were re-plotted with grouping by mouse over time (Fig. S5).

**Figure 4:**
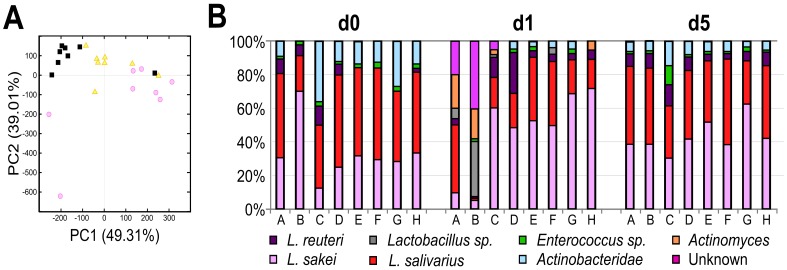
LAB-TRFLP analysis of fecal microbiota in mice following a combined facility/cage change. A) Principal coordinate analysis of LAB-TRFLP data at days 0, 1 and 5 in the control group (group 3, n = 8). Each data point represents a single mouse specimen at days 0 (pink circles), 1 (black squares), and 5 (yellow triangles). B) Taxonomic plot of lactic acid bacterial profiles on days 0, 1 and 5. Operational taxonomic units were assigned based on an *in silico* digest-database to estimate relative proportions of the *Lactobacillales* present in the samples. Each bar represents a single mouse specimen. Mice A, B, G, H were males; C, D, E, F were females.

To further elucidate the dramatic quantitative changes in *Lactobacillales*, we examined the composition of this order using focused LAB-TRFLP [37]. We sought to determine if the lactic acid bacterial communities were resilient following the dramatically decreased abundance observed at day 1. Indeed, a PCoA of the LAB-TRFLP data (Fig. 4A) showed variation in the *Lactobacillales* population at day 1, with a return to a diffuse pattern at day 5. The taxonomic plots showed subtle differences in the relative proportion of the most common species - *Lactobacillus reuteri*, *Lactobacillus salivarius* and *Lactobacillus sakei*, and increases in species that had previously not been detectable (Fig. 4B). This suggests that the population of lactic acid bacteria was initially sensitive to the influences imposed by the facility/cage change, but were quite resilient - recovering to relative proportions approximating baseline by day 5.

**Figure 5:**
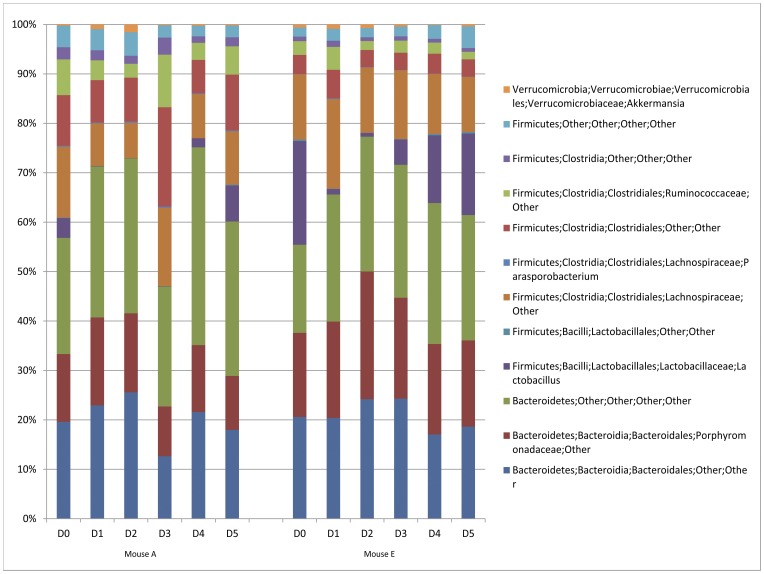
Genomic sequence analysis of fecal microbiota in mice following a combined facility/cage change. Fecal samples from two mice in the control (group 3) cohort were collected daily prior (D0) to and 5 days (D1–D5) following cage change and analyzed by NGS. Taxonomic plots derived from the NGS data from V4 rDNA are plotted with *y*-axis representing relative OTU abundance (left, mouse A; right, mouse E, both as labeled in Fig. 2 and 3).

To more completely elucidate the microbiota composition in these mice over the course of the 5 days, NGS was performed on 2 individual mice (A and E) in the control group (group 3). Taxonomic plots (Fig. 5) of the sequence data (Table S3) are presented for each mouse. Similar to our previous assessments, a diverse bacterial population was observed for each mouse at day 0 and 5 (Fig. 5). In addition a virtual disappearance of the Lactobacillales group was apparent at day 1, with its abundance returning during days 2–4. These results support the interpretations from the TRFLP and qPCR data. (Figs. 3 and S1A).

**Figure 6:**
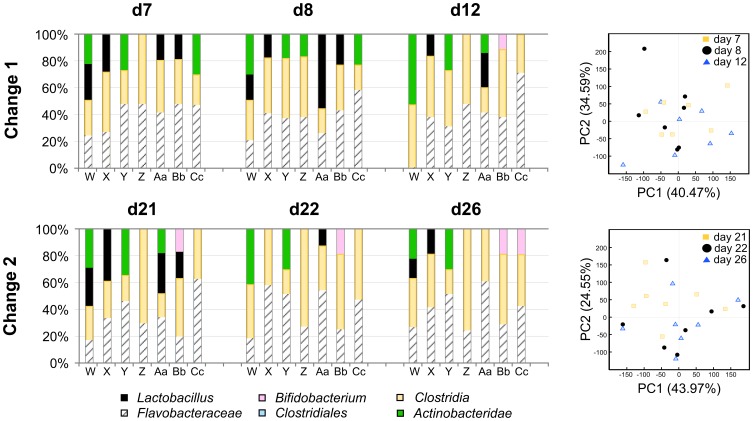
Eubacterial-TRFLP analysis of fecal microbiota in mice following two routine cage changes. Taxonomic profile (left) and principal coordinate analysis (right) of TRFLP data. Operational taxonomic units were assigned based on an *in silico* digest-database to estimate relative proportions of each major bacterial group present in the samples. Each bar represents a single mouse specimen prior to cage change (d7, d21), 1 day after cage change (d8, d22), and 5 days after cage change (d12, d26). Each PCoA data point represents these individual specimens prior to cage change (yellow squares, d7, d21), 1 day after cage change (black circles, d8, d22), and 5 days after cage change (blue triangles, d12, d26). Mice X, Y, Bb, and Cc were males; W, Z, and Aa were females.

Finally, the composition of the microbiota in a group of mice that experienced a cage change, but without a change of housing facility was assessed by Eubacterial-TRFLP analysis of stool microbiota. A cohort of mice (group 5, n = 7), like before were transferred from a SPF barrier facility to a conventional housing facility and maintained on corncob bedding and Harlan Teklad 2918 diet for 37 days. Fresh stool was sampled daily and routine cage change was performed every seven days, per institutional policy. In this experiment, mice were allowed to acclimate after the day 0 facility change prior to assessing the effects of the cage change at day 7 and 22. Eubacterial-TRFLP analysis of individual taxonomic groups in stool sampled just prior to, and then 1- and 5-days following the routine changes showed no statistically significant differences in microbiota (Fig. 6). PCoA of this cohort of mice showed a diffuse scatter pattern at each time-point, and the taxonomic plot indicates a similar profile of each individual on all days. While our data cannot rule-out a significant (but subtle) difference in the days immediately following a cage change, the profound changes that accompanied the combined facility transfer/cage change (Figs. 1, 2, 3, S1, and S2) were not observed in the acclimated mice.

## Discussion

A healthy microbiota is essential for fitness of the host [2]–[4]; conversely, dysbiosis can promote illness by increasing the susceptibility to and/or persistence of disease [13], [17]. Mouse models have been at the forefront of research to elucidate the mechanisms that link the composition of the microbiota with normal physiology or disease [18], [20]–[30]. Husbandry practices can potentially impact the composition of microbiota, and hence introduce variables into research studies and affect experimental outcomes [34]. Although often mentioned in the description of research methodology as an accepted assumption, surprisingly little has been published to systematically test the importance of these environmental variables on microbiota [51]. In this study, our data provide a glimpse into the significant changes of the intestinal microbiota that result from a simple “routine” intracampus facility change to a conventional animal facility.

We observed a rather profound difference in the composition of the colonizing microbial community one day after the short facility transfer with its attendant cage change that gradually returned to approximate baseline composition by day 5. These data identify a previously unrecognized transient alteration in intestinal microbiota following the routine relocation and change of a mouse’s cage. The findings suggest that without an acclimation period, a “routine” facility change has potential importance in numerous scenarios, e.g. sampling of microbiota following introduction of experimental variables, timing of pathogen challenges, time-course of investigations probing metabolic dynamics, and perhaps analysis of behavioral phenotypes.

The two variables that we tested at the outset - bedding and standard chow - showed no detectable effect on microbiota over the relatively short five-day time course in this study. It remains possible that these husbandry changes could have a significant effect on composition profiles over a longer time span. Likewise, it is possible that changes in abundance of members of the microbiota could have escaped our detection. Nevertheless, we did observe microbiota composition changes by day 5 upon switching mice to a high fat diet, consistent with previous reports [20], [48], [49], [52]. Rather than further pursuing the possible nuances of these hypotheses, we focused attention to the striking aftereffects of a facility and cage change.

From behavioral observations, mouse biologists have long recognized that change in environment is a stressful event for laboratory rodents. Because mice rely heavily on odors for communication, territorial marking, and control of sexual and aggressive behaviors [53], disrupting their environment has consequence on olfactory cues. In addition, coprophagy is necessary to provide many essential nutrients, including B vitamins, synthesized by bacteria in the distal intestine [54]. Together, the importance of olfaction and coprophagy in normal mouse biology suggests why the environmental upheaval imposed by facility change might have physiological repercussions. Cage changes, inter-facility transport, and even simple handling of mice resulted in transient elevations of corticosterone levels [55], [56], consistent with these activities as being stressful. On the other hand, changing cages helps avoid toxic in-cage accumulation of ammonia and carbon dioxide, as well as minimizing noxious odors [57]. Thus, guidelines governing the frequency of changing cages include consideration of hygiene, health of the animals, health of facility personnel and costs [58]. Fortunately for ongoing experimental protocols in many laboratories, our data did not discern a microbiota change upon simple cage change after mice were acclimated to their new facility, although the possibility of a subtle, but significant, change in colonizing microbes should not disregarded in all situations.

Looking at the taxonomic data, the evidence indicates a significant decrease in *Lactobacillales* from the microbiota within the first 24 hours of a combined facility/cage change. Several other changes in the microbiota composition were also evident. After the initial shift in microbiota, there was a return to a composition that resembled baseline by day 5. This resilience supports that a rest period of approximately five days may be adequate for laboratory mice to acclimate following intracampus facility change. In addition, the results reported here suggest that the composition of the microbiota could be a useful non-invasive parameter to refine husbandry practices to minimize impact, optimizing both experimental protocols and evidence-based guidelines for animal care.

## Supporting Information

Figure S1 **Analysis of stool microbiota following facility and cage change in an independent cohort of mice (group 5).** Fresh fecal samples were collected on d0, d1, and d5 from individual mice. Day 0 denotes baseline samples obtained just prior to transfer from an SPF facility to a conventional housing facility. Upon transfer, mice were placed in a fresh cage, but maintained on the same type of food and bedding (corncob and Teklad 2918). Day 1 is 24 hours after the combined facility transfer/cage change. A) Taxonomic profile of TRFLP data. Operational taxonomic units were assigned based on an *in silico* digest-database to estimate relative proportions of each major bacterial group present in the samples. Each bar represents a single mouse specimen. B) Principal coordinate analysis of Eubacterial-TRFLP data. Each PCoA data point represents these individual specimens prior to cage change (yellow square), 1 day after cage change (black circles), and 5 days after cage change (blue triangles). Mice X, Y, Bb, and Cc were males; W, Z, and Aa were females.
(TIF)

Figure S2 **Quantitative PCR analysis of stool microbiota of individual mice in bedding, food and control cohorts (groups 1–3).** A) qPCR analysis of stool microbiota of individual mice in control cohort (group 3) before and one day after cage change. DNA isolated from stool of each mouse in group 3 (shown in Fig. 1) at day 0 and day 1 was analyzed by quantitative PCR as described in the methods. The y-axis represents the quantitative estimates for each bacterial group normalized to the total abundance of bacteria from the kingdom-specific *Eubacteria* assay. For each mouse at each time point, the normalized value for each bacterial group was compared by paired t-test. *P*-values are indicated for each analysis. B, C) Quantitative PCR analysis of stool microbiota of mice in bedding, food and control cohorts (groups 1–3) at day 5. DNA isolated from stool on day 5 of each mouse shown in Fig. 1 was analyzed by quantitative PCR as described in the methods. B) Quantitative comparison of five bacterial groups. For each cohort of mice, the average normalized values (+/− SD) for each bacterial group were compared to the control group by Student’s t-test. No significant differences were noted between cohorts for any bacterial group. C) Graphical representation of microbiota composition for each cohort of mice.
(TIF)

Figure S3 **Principal coordinate analysis of Eubacterial-TRFLP data for mice switched to a high fat diet.** A. Control mice (group 5) were maintained without change of food or bedding (Teklad 2918 and corncob, respectively). Each data point represents a single mouse specimen at day 0 (gray) and day 5 (black). B. Experimental mice (group 4) were switched to a high fat diet after stool specimen collection on day 0. Each data point represents a single mouse specimen of mice were housed on corncob (closed circle and squares) or Carefresh (open circle and squares), at day 0 (gray) and day 5 (black).
(TIF)

Figure S4 **Effect of high fat diet on microbiota composition determined by quantitative PCR analysis.** Mice were maintained for 5 days on either high fat diet (group 4, n = 8) or regular diet (group 5, n = 7) and DNA isolated from stool of each mouse was analyzed by quantitative PCR as described in the methods. The y-axis represents the quantitative estimates for each bacterial group normalized to the total abundance of bacteria from the kingdom-specific *Eubacteria* assay (+/− SD). *P*-values are indicated for each analysis (Student’s t-test).
(TIF)

Figure S5 **Taxonomic plot of eubacterial-TRFLP data of fecal microbiota in mice following a combined facility/cage change (alternative display of data organized by individual mice).** A reorganization of data shown in Fig. 3 to better view changes in individual mice daily from day 0 to day 5. The facility/cage change occurred on day 0 after stool was sampled (see Fig. 1A for timeline). DNA isolated from the feces of each mouse in the control group (group 3, n = 8) was analyzed by TRFLP as described in the methods. Mice A, B, G, H were males; C, D, E, F were females. Note that over time, the group of *Lactobacillus* initially lost at day 1, returns to the population profile in 5 of 8 mice by day 4, and 6 of 8 mice by day 5.
(TIF)

Table S1 **Oligonucleotide primer sequences and annealing temperatures for qPCR assays.**
(PDF)

Table S2 **NGS primer and barcode sequences.**
(PDF)

Table S3 **NGS taxonomic data of fecal microbiota in mice following a combined facility/cage change.**
(PDF)

## References

[1] Costello EK, Lauber CL, Hamady M, Fierer N, Gordon JI, et al (2009) Bacterial community variation in human body habitats across space and time. Science 326: 1694–1697. 19892944 10.1126/science.1177486 PMC3602444

[2] Hooper LV (2004) Bacterial contributions to mammalian gut development. Trends Microbiol 12: 129–134. 15001189 10.1016/j.tim.2004.01.001

[3] Ley RE, Lozupone CA, Hamady M, Knight R, Gordon JI (2008) Worlds within worlds: evolution of the vertebrate gut microbiota. Nat Rev Microbiol 6: 776–788. 18794915 10.1038/nrmicro1978 PMC2664199

[4] Zaneveld J, Turnbaugh PJ, Lozupone C, Ley RE, Hamady M, et al (2008) Host-bacterial coevolution and the search for new drug targets. Curr Opin Chem Biol 12: 109–114. 18280814 10.1016/j.cbpa.2008.01.015 PMC2348432

[5] Handelsman J (2004) Metagenomics: application of genomics to uncultured microorganisms. Microbiol Mol Biol Rev 68: 669–685. 15590779 10.1128/MMBR.68.4.669-685.2004 PMC539003

[6] Gordon JI, Klaenhammer TR (2011) A rendezvous with our microbes. Proc Natl Acad Sci U S A 108 Suppl 1 4513–4515. 21406595 10.1073/pnas.1101958108 PMC3063591

[7] Reyes A, Haynes M, Hanson N, Angly FE, Heath AC, et al (2010) Viruses in the faecal microbiota of monozygotic twins and their mothers. Nature 466: 334–338. 20631792 10.1038/nature09199 PMC2919852

[8] Samuel BS, Gordon JI (2006) A humanized gnotobiotic mouse model of host-archaeal-bacterial mutualism. Proc Natl Acad Sci U S A 103: 10011–10016. 16782812 10.1073/pnas.0602187103 PMC1479766

[9] Scupham AJ, Presley LL, Wei B, Bent E, Griffith N, et al (2006) Abundant and Diverse Fungal Microbiota in the Murine Intestine. Applied and Environmental Microbiology 72: 793–801. 16391120 10.1128/AEM.72.1.793-801.2006 PMC1352209

[10] LaTuga MS, Ellis JC, Cotton CM, Goldberg RN, Wynn JL, et al (2011) Beyond bacteria: a study of the enteric microbial consortium in extremely low birth weight infants. PLoS ONE 6: e27858. 22174751 10.1371/journal.pone.0027858 PMC3234235

[11] Palmer C, Bik EM, DiGiulio DB, Relman DA, Brown PO (2007) Development of the human infant intestinal microbiota. PLoS Biol 5: e177. 17594176 10.1371/journal.pbio.0050177 PMC1896187

[12] Hooper LV, Macpherson AJ (2010) Immune adaptations that maintain homeostasis with the intestinal microbiota. Nat Rev Immunol 10: 159–169. 20182457 10.1038/nri2710

[13] Chow J, Lee SM, Shen Y, Khosravi A, Mazmanian SK (2010) Host-Bacterial Symbiosis in Health and Disease. Mucosal Immunity. 243–274. 10.1016/B978-0-12-381300-8.00008-3 PMC3152488 21034976

[14] Ley RE, Hamady M, Lozupone C, Turnbaugh PJ, Ramey RR, et al (2008) Evolution of mammals and their gut microbes. Science 320: 1647–1651. 18497261 10.1126/science.1155725 PMC2649005

[15] O’Hara AM, Shanahan F (2006) The gut flora as a forgotten organ. EMBO Rep 7: 688–693. 16819463 10.1038/sj.embor.7400731 PMC1500832

[16] Gonzalez A, Stombaugh J, Lozupone C, Turnbaugh PJ, Gordon JI, et al (2011) The mind-body-microbial continuum. Dialogues Clin Neurosci 13: 55–62. 21485746 10.31887/DCNS.2011.13.1/agonzalez PMC3139398

[17] Chow J, Tang H, Mazmanian SK (2011) Pathobionts of the gastrointestinal microbiota and inflammatory disease. Curr Opin Immunol 23: 473–480. 21856139 10.1016/j.coi.2011.07.010 PMC3426444

[18] Sekirov I, Finlay BB (2009) The role of the intestinal microbiota in enteric infection. J Physiol 587: 4159–4167. 19491248 10.1113/jphysiol.2009.172742 PMC2754356

[19] Packey CD, Sartor RB (2009) Commensal bacteria, traditional and opportunistic pathogens, dysbiosis and bacterial killing in inflammatory bowel diseases. Current Opinion in Infectious Diseases 22: 292–301. 19352175 10.1097/QCO.0b013e32832a8a5d PMC2763597

[20] Turnbaugh PJ, Ley RE, Mahowald MA, Magrini V, Mardis ER, et al (2006) An obesity-associated gut microbiome with increased capacity for energy harvest. Nature 444: 1027–1031. 17183312 10.1038/nature05414

[21] Dumas M-E, Barton RH, Toye A, Cloarec O, Blancher C, et al (2006) Metabolic profiling reveals a contribution of gut microbiota to fatty liver phenotype in insulin-resistant mice. Proceedings of the National Academy of Sciences 103: 12511–12516. 10.1073/pnas.0601056103 PMC1567909 16895997

[22] Wen L, Ley RE, Volchkov PY, Stranges PB, Avanesyan L, et al (2008) Innate immunity and intestinal microbiota in the development of Type 1 diabetes. Nature 455: 1109–1113. 18806780 10.1038/nature07336 PMC2574766

[23] Nell S, Suerbaum S, Josenhans C (2010) The impact of the microbiota on the pathogenesis of IBD: lessons from mouse infection models. Nat Rev Microbiol 8: 564–577. 20622892 10.1038/nrmicro2403

[24] Backhed F (2011) Programming of host metabolism by the gut microbiota. Ann Nutr Metab 58 Suppl 2 44–52. 21846980 10.1159/000328042

[25] Tilg H, Kaser A (2011) Gut microbiome, obesity, and metabolic dysfunction. Journal of Clinical Investigation 121: 2126–2132. 21633181 10.1172/JCI58109 PMC3104783

[26] Candela M, Guidotti M, Fabbri A, Brigidi P, Franceschi C, et al (2011) Human intestinal microbiota: cross-talk with the host and its potential role in colorectal cancer. Crit Rev Microbiol 37: 1–14. 20874522 10.3109/1040841X.2010.501760

[27] Plottel CS, Blaser MJ (2011) Microbiome and Malignancy. Cell Host & Microbe 10: 324–335. 22018233 10.1016/j.chom.2011.10.003 PMC3264051

[28] Koren O, Spor A, Felin J, Fak F, Stombaugh J, et al (2011) Human oral, gut, and plaque microbiota in patients with atherosclerosis. Proc Natl Acad Sci U S A 108 Suppl 1 4592–4598. 20937873 10.1073/pnas.1011383107 PMC3063583

[29] Mathis D, Benoist C (2012) The influence of the microbiota on type-1 diabetes: on the threshold of a leap forward in our understanding. Immunological Reviews 245: 239–249. 22168424 10.1111/j.1600-065X.2011.01084.x

[30] Hill DA, Siracusa MC, Abt MC, Kim BS, Kobuley D, et al.. (2012) Commensal bacteria-derived signals regulate basophil hematopoiesis and allergic inflammation. Nat Med. 10.1038/nm.2657 PMC3321082 22447074

[31] Malakoff D (2000) The rise of the mouse, biomedicine’s model mammal. Science 288: 248–+. 10.1126/science.288.5464.248 10777401

[32] Schellinck HM, Cyr DP, Brown RE (2010) How Many Ways Can Mouse Behavioral Experiments Go Wrong? Confounding Variables in Mouse Models of Neurodegenerative Diseases and How to Control Them. In: Advances in the Study of Behavior, Vol Brockmann HJ, Roper TJ, Naguib M, WynneEdwards KE, Mitani JC, et al, editors. editors. 41: 255–366.

[33] Gurfein BT, Stamm AW, Bacchetti P, Dallman MF, Nadkarni NA, et al (2012) The calm mouse: an animal model of stress reduction. Mol Med 18: 606–617. 22398685 10.2119/molmed.2012.00053 PMC3388136

[34] Bleich A, Hansen AK (2012) Time to include the gut microbiota in the hygienic standardisation of laboratory rodents. Comp Immunol Microbiol Infect Dis 35: 81–92. 22257867 10.1016/j.cimid.2011.12.006

[35] Salzman NH, Hung K, Haribhai D, Chu H, Karlsson-Sjoberg J, et al (2010) Enteric defensins are essential regulators of intestinal microbial ecology. Nat Immunol 11: 76–83. 19855381 10.1038/ni.1825 PMC2795796

[36] Bokulich NA, Bamforth CW, Mills DA (2012) Brewhouse-resident microbiota are responsible for multi-stage fermentation of american coolship ale. PLoS ONE 7: e35507. 22530036 10.1371/journal.pone.0035507 PMC3329477

[37] Bokulich NA, Mills DA (2012) Differentiation of mixed lactic acid bacteria communities in beverage fermentations using targeted terminal restriction fragment length polymorphism. Food Microbiol 31: 126–132. 22475950 10.1016/j.fm.2012.02.007

[38] Bokulich NA, Joseph CM, Allen G, Benson AK, Mills DA (2012) Next-generation sequencing reveals significant bacterial diversity of botrytized wine. PLoS ONE 7: e36357. 22563494 10.1371/journal.pone.0036357 PMC3341366

[39] Caporaso JG, Lauber CL, Walters WA, Berg-Lyons D, Lozupone CA, et al (2011) Global patterns of 16S rRNA diversity at a depth of millions of sequences per sample. Proc Natl Acad Sci U S A 108 Suppl 1 4516–4522. 20534432 10.1073/pnas.1000080107 PMC3063599

[40] Abdo Z, Schuette UM, Bent SJ, Williams CJ, Forney LJ, et al (2006) Statistical methods for characterizing diversity of microbial communities by analysis of terminal restriction fragment length polymorphisms of 16S rRNA genes. Environ Microbiol 8: 929–938. 16623749 10.1111/j.1462-2920.2005.00959.x

[41] Shyu C, Soule T, Bent SJ, Foster JA, Forney LJ (2007) MiCA: a web-based tool for the analysis of microbial communities based on terminal-restriction fragment length polymorphisms of 16S and 18S rRNA genes. Microb Ecol 53: 562–570. 17406775 10.1007/s00248-006-9106-0

[42] Cole JR, Chai B, Farris RJ, Wang Q, Kulam-Syed-Mohideen AS, et al (2007) The ribosomal database project (RDP-II): introducing myRDP space and quality controlled public data. Nucleic Acids Res 35: D169–172. 17090583 10.1093/nar/gkl889 PMC1669760

[43] Cole JR, Wang Q, Cardenas E, Fish J, Chai B, et al (2009) The Ribosomal Database Project: improved alignments and new tools for rRNA analysis. Nucleic Acids Res 37: D141–145. 19004872 10.1093/nar/gkn879 PMC2686447

[44] Caporaso JG, Kuczynski J, Stombaugh J, Bittinger K, Bushman FD, et al (2010) QIIME allows analysis of high-throughput community sequencing data. Nat Methods 7: 335–336. 20383131 10.1038/nmeth.f.303 PMC3156573

[45] Edgar RC (2010) Search and clustering orders of magnitude faster than BLAST. Bioinformatics 26: 2460–2461. 20709691 10.1093/bioinformatics/btq461

[46] Wang Q, Garrity GM, Tiedje JM, Cole JR (2007) Naive Bayesian classifier for rapid assignment of rRNA sequences into the new bacterial taxonomy. Appl Environ Microbiol 73: 5261–5267. 17586664 10.1128/AEM.00062-07 PMC1950982

[47] DeSantis TZ, Hugenholtz P, Larsen N, Rojas M, Brodie EL, et al (2006) Greengenes, a chimera-checked 16S rRNA gene database and workbench compatible with ARB. Appl Environ Microbiol 72: 5069–5072. 16820507 10.1128/AEM.03006-05 PMC1489311

[48] Hildebrandt MA, Hoffmann C, Sherrill-Mix SA, Keilbaugh SA, Hamady M, et al.. (2009) High-fat diet determines the composition of the murine gut microbiome independently of obesity. Gastroenterology 137: 1716–1724 e1711–1712. 10.1053/j.gastro.2009.08.042 PMC2770164 19706296

[49] Ley RE, Backhed F, Turnbaugh P, Lozupone CA, Knight RD, et al (2005) Obesity alters gut microbial ecology. Proc Natl Acad Sci U S A 102: 11070–11075. 16033867 10.1073/pnas.0504978102 PMC1176910

[50] Turnbaugh PJ, Ridaura VK, Faith JJ, Rey FE, Knight R, et al (2009) The effect of diet on the human gut microbiome: a metagenomic analysis in humanized gnotobiotic mice. Sci Transl Med 1: 6ra14. 10.1126/scitranslmed.3000322 PMC2894525 20368178

[51] Hufeldt MR, Nielsen DS, Vogensen FK, Midtvedt T, Hansen AK (2010) Variation in the gut microbiota of laboratory mice is related to both genetic and environmental factors. Comp Med 60: 336–347. 21262117 PMC2958200

[52] Turnbaugh PJ, Backhed F, Fulton L, Gordon JI (2008) Diet-induced obesity is linked to marked but reversible alterations in the mouse distal gut microbiome. Cell Host Microbe 3: 213–223. 18407065 10.1016/j.chom.2008.02.015 PMC3687783

[53] Hurst JL, Beynon RJ (2004) Scent wars: the chemobiology of competitive signalling in mice. Bioessays 26: 1288–1298. 15551272 10.1002/bies.20147

[54] Soave O, Brand CD (1991) Coprophagy in animals - a review. Cornell Veterinarian 81: 357–364. 1954740

[55] Tuli JS, Smith JA, Morton DB (1995) Stress measurements in mice after transportation. Lab Anim 29: 132–138. 7602999 10.1258/002367795780740249

[56] Rasmussen S, Miller MM, Filipski SB, Tolwani RJ (2011) Cage Change Influences Serum Corticosterone and Anxiety-Like Behaviors in the Mouse. Journal of the American Association for Laboratory Animal Science 50: 479–483. 21838975 PMC3148651

[57] Reeb-Whitaker CK, Paigen B, Beamer WG, Bronson RT, Churchill GA, et al (2001) The impact of reduced frequency of cage changes on the health of mice housed in ventilated cages. Laboratory Animals 35: 58–73. 11201289 10.1258/0023677011911381

[58] Jennings M, Batchelor GR, Brain PF, Dick A, Elliott H, et al (1998) Refining rodent husbandry: the mouse - Report of the Rodent Refinement Working Party. Laboratory Animals 32: 233–259. 9718472 10.1258/002367798780559301

