# Correction: Routine Habitat Change: A Source of Unrecognized Transient Alteration of Intestinal Microbiota in Laboratory Mice

**DOI:** 10.1371/annotation/7e4429bf-b5a6-43d6-8b03-1457e01450e3

**Published:** 2013-10-08

**Authors:** Betty W. Ma, Nicholas A. Bokulich, Patricia A. Castillo, Anchasa Kananurak, Mark A. Underwood, David A. Mills, Charles L. Bevins

In the second sentence of the “Next Generation Sequencing (NGS) Library Construction” section, the sequence for primer F515 appears incorrectly in the text. The sentence should read: “Briefly, the V4 domain of bacterial 16S rDNA was amplified using primers F515 (5′-GTGCCAGCMGCCGCGGTAA-3′).”

