# Supplementary figures and images for: Routine Habitat Change: A Source of Unrecognized Transient Alteration of Intestinal Microbiota in Laboratory Mice
Source: PLoS One. 2012 Oct 17;7(10):e47416. doi: 10.1371/journal.pone.0047416 (PMC3474821; doi:10.1371/journal.pone.0047416)

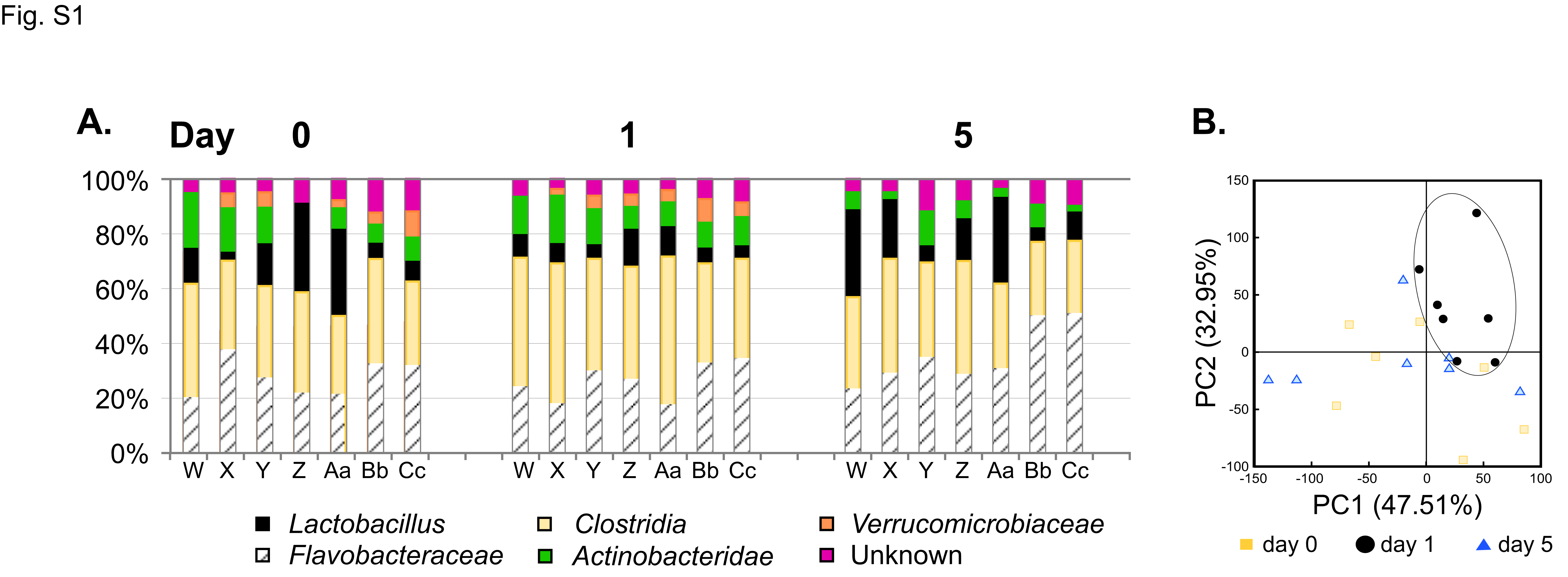

Supplement: Figure S1 — Analysis of stool microbiota following facility and cage change in an independent cohort of mice (group 5). Fresh fecal samples were collected on d0, d1, and d5 from individual mice. Day 0 denotes baseline samples obtained just prior to transfer from an SPF facility to a conventional housing facility. Upon transfer, mice were placed in a fresh cage, but maintained on the same type of food and bedding (corncob and Teklad 2918). Day 1 is 24 hours after the combined facility transfer/cage change. A) Taxonomic profile of TRFLP data. Operational taxonomic units were assigned based on an in silico digest-database to estimate relative proportions of each major bacterial group present in the samples. Each bar represents a single mouse specimen. B) Principal coordinate analysis of Eubacterial-TRFLP data. Each PCoA data point represents these individual specimens prior to cage change (yellow square), 1 day after cage change (black circles), and 5 days after cage change (blue triangles). Mice X, Y, Bb, and Cc were males; W, Z, and Aa were females. (TIF) [file pone.0047416.s001.tif]

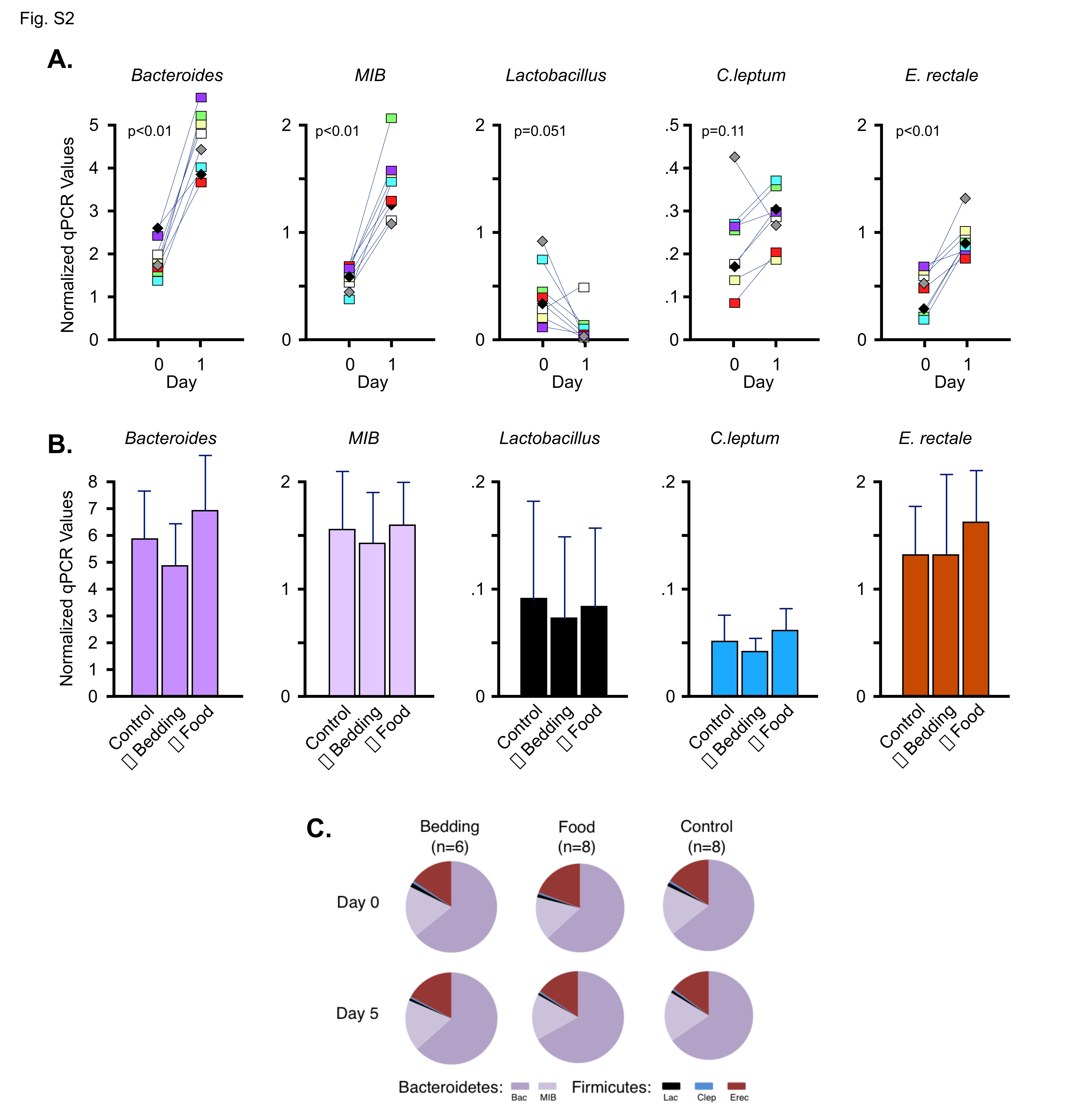

Supplement: Figure S2 — Quantitative PCR analysis of stool microbiota of individual mice in bedding, food and control cohorts (groups 1–3). A) qPCR analysis of stool microbiota of individual mice in control cohort (group 3) before and one day after cage change. DNA isolated from stool of each mouse in group 3 (shown in Fig. 1) at day 0 and day 1 was analyzed by quantitative PCR as described in the methods. The y-axis represents the quantitative estimates for each bacterial group normalized to the total abundance of bacteria from the kingdom-specific Eubacteria assay. For each mouse at each time point, the normalized value for each bacterial group was compared by paired t-test. P-values are indicated for each analysis. B, C) Quantitative PCR analysis of stool microbiota of mice in bedding, food and control cohorts (groups 1–3) at day 5. DNA isolated from stool on day 5 of each mouse shown in Fig. 1 was analyzed by quantitative PCR as described in the methods. B) Quantitative comparison of five bacterial groups. For each cohort of mice, the average normalized values (+/− SD) for each bacterial group were compared to the control group by Student’s t-test. No significant differences were noted between cohorts for any bacterial group. C) Graphical representation of microbiota composition for each cohort of mice. (TIF) [file pone.0047416.s002.tif]

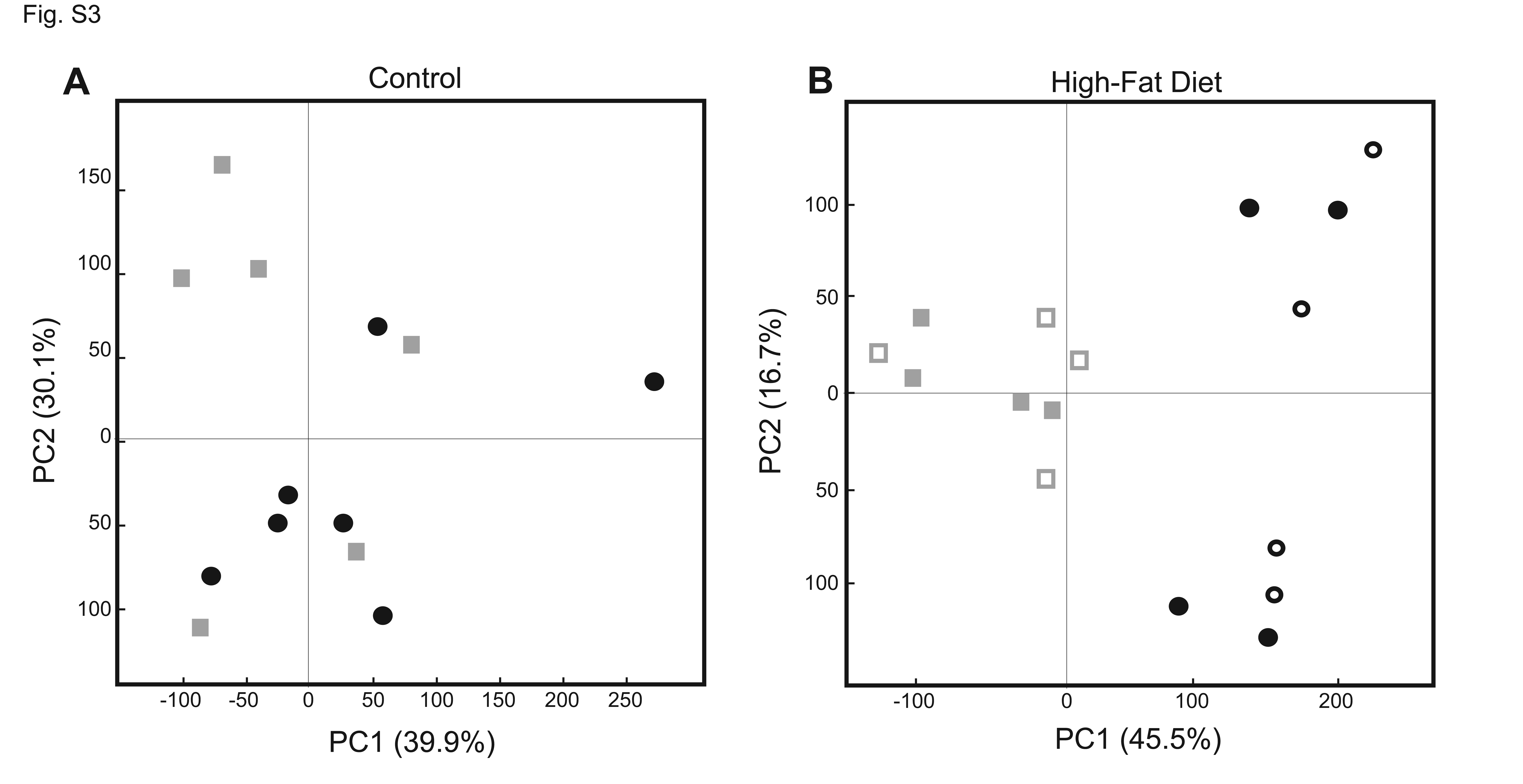

Supplement: Figure S3 — Principal coordinate analysis of Eubacterial-TRFLP data for mice switched to a high fat diet. A. Control mice (group 5) were maintained without change of food or bedding (Teklad 2918 and corncob, respectively). Each data point represents a single mouse specimen at day 0 (gray) and day 5 (black). B. Experimental mice (group 4) were switched to a high fat diet after stool specimen collection on day 0. Each data point represents a single mouse specimen of mice were housed on corncob (closed circle and squares) or Carefresh (open circle and squares), at day 0 (gray) and day 5 (black). (TIF) [file pone.0047416.s003.tif]

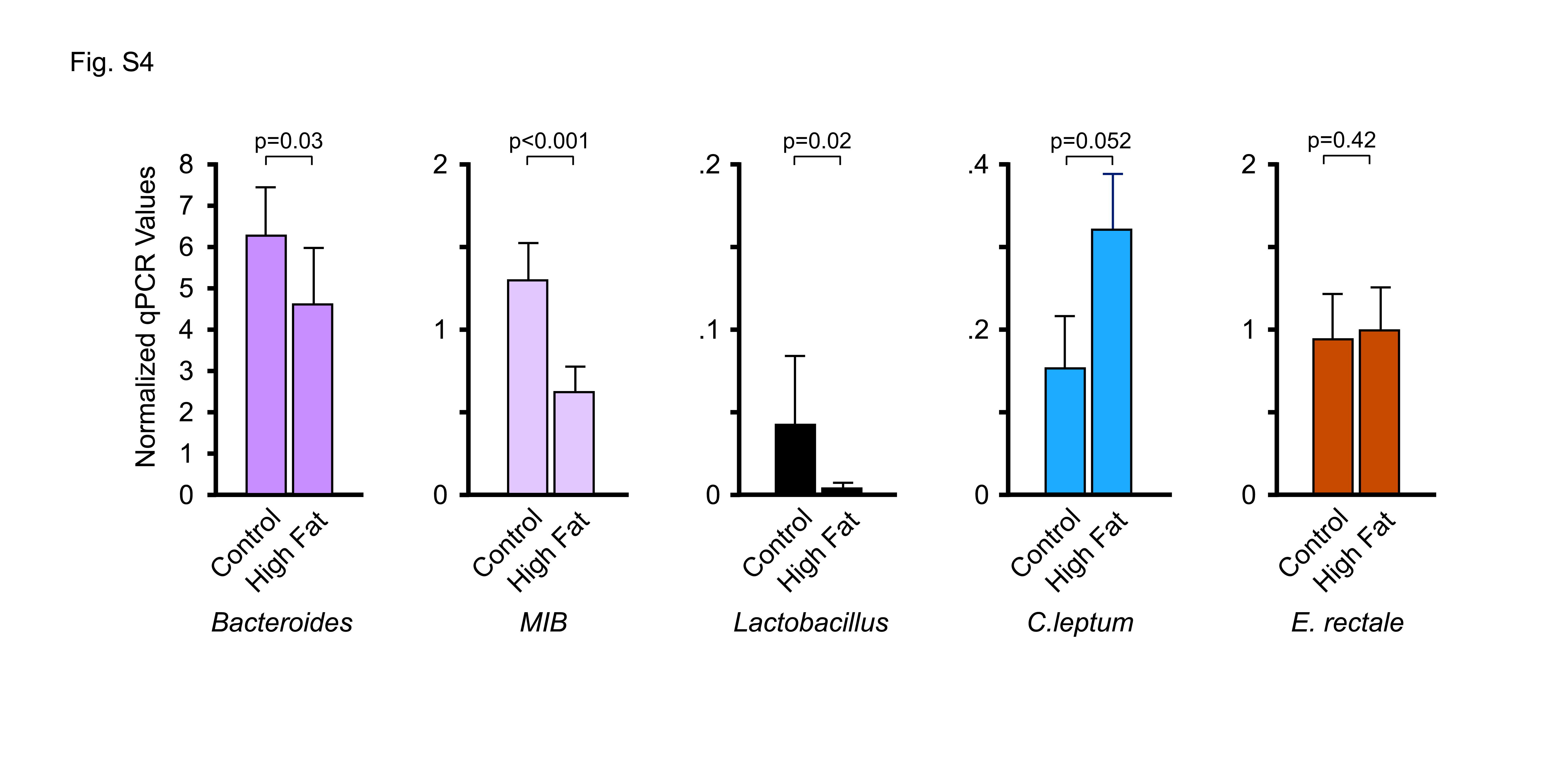

Supplement: Figure S4 — Effect of high fat diet on microbiota composition determined by quantitative PCR analysis. Mice were maintained for 5 days on either high fat diet (group 4, n = 8) or regular diet (group 5, n = 7) and DNA isolated from stool of each mouse was analyzed by quantitative PCR as described in the methods. The y-axis represents the quantitative estimates for each bacterial group normalized to the total abundance of bacteria from the kingdom-specific Eubacteria assay (+/− SD). P-values are indicated for each analysis (Student’s t-test). (TIF) [file pone.0047416.s004.tif]

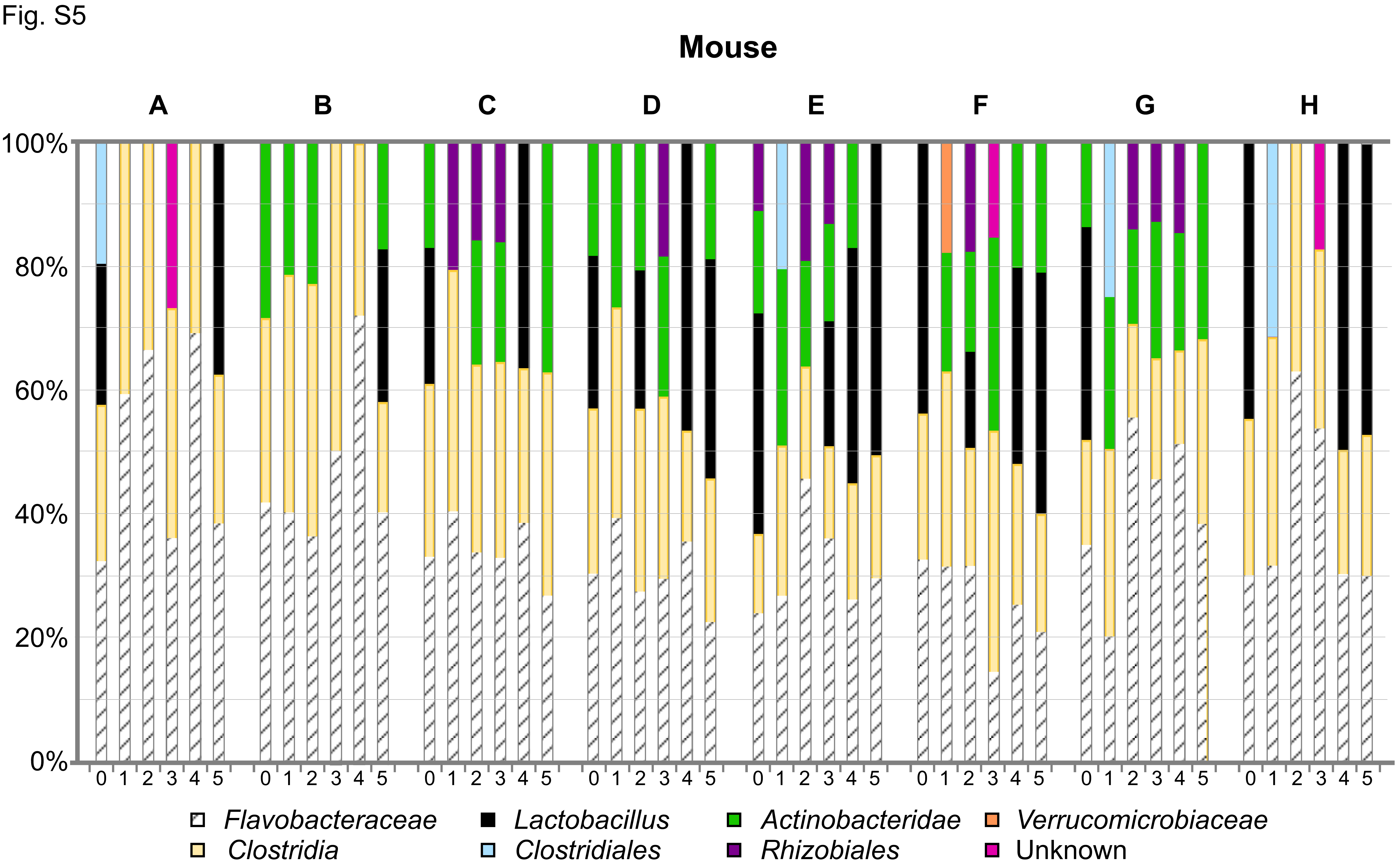

Supplement: Figure S5 — Taxonomic plot of eubacterial-TRFLP data of fecal microbiota in mice following a combined facility/cage change (alternative display of data organized by individual mice). A reorganization of data shown in Fig. 3 to better view changes in individual mice daily from day 0 to day 5. The facility/cage change occurred on day 0 after stool was sampled (see Fig. 1A for timeline). DNA isolated from the feces of each mouse in the control group (group 3, n = 8) was analyzed by TRFLP as described in the methods. Mice A, B, G, H were males; C, D, E, F were females. Note that over time, the group of Lactobacillus initially lost at day 1, returns to the population profile in 5 of 8 mice by day 4, and 6 of 8 mice by day 5. (TIF) [file pone.0047416.s005.tif]
