# Supplementary material for: Routine Habitat Change: A Source of Unrecognized Transient Alteration of Intestinal Microbiota in Laboratory Mice
Source: PLoS One. 2012 Oct 17;7(10):e47416. doi: 10.1371/journal.pone.0047416 (PMC3474821; doi:10.1371/journal.pone.0047416)
Supplement: Table S1 — Oligonucleotide primer sequences and annealing temperatures for qPCR assays. (PDF) [file pone.0047416.s006.pdf]

Table S1. Oligonucleotide primer sequences and annealing temperatures for qPCR assays.

Assay	Primers (5'-3')	Annealing temperature (ºF)
Eubacteria	F: ACTCCTACGGGAGGCAGCAGT<br>R: ATTACCGCGGCTGCTGGC	62
Bacteroides	F: GGTTCTGAGAGGAGGTCCC<br>R: CTGCCTCCCGTAGGAGT	60
Mouse intestinal bacteroides (MIB)	F: CCAGCAGCCGCGGTAATA<br>R: CGCATTCCGCATACTTCTC	58
Lactobacillus	F: AGCAGTAGGGAATCTTCCA<br>R: CACCGCTACACATGGAG	58
C. leptum	F: GTTGACAAAACGGAGGAAGG<br>R: GACGGGCGGTGTGTACAA	60
E. rectale	F: ACTCCTACGGGAGGCAGC<br>R: CTTCTTAGTCAGGTACCGTCAT	61
