# Supplementary material for: Routine Habitat Change: A Source of Unrecognized Transient Alteration of Intestinal Microbiota in Laboratory Mice
Source: PLoS One. 2012 Oct 17;7(10):e47416. doi: 10.1371/journal.pone.0047416 (PMC3474821; doi:10.1371/journal.pone.0047416)
Supplement: Table S2 — NGS primer and barcode sequences. (PDF) [file pone.0047416.s007.pdf]

**Suppl. Table 2.** NGS primer and barcode sequences. Underlined regions indicate Illumina adapter sequence. Boldface text indicates V4 PCR primer region, preceded by linker sequence. Poly-N string in forward primer denotes barcode sequence.

| | |
|---|---|
| Forward Primer | AATGATACGGCGACCACCGAGATCTACACTCTTTCCCTACACGACGCTCTTCCGATCTNNNNNNNNGT**GTGCCAGCMGCCGCGGTAA** |
| Reverse Primer | CAAGCAGAAGACGGCATACGAGATCGGTCTCGGCATTCCTGCTGAACCGCTCTTCCGATCTCC**GGACTACHVGGGTWTCTAAT** |
| Barcodes | **AGGTACGA, AGGTAGCA, AGGTAGGT, AGGTCAAG, AGGTCATC, AGGTCTAC, AGGTCTTG, AGGTGAAC, AGGTGATG, AGGTGTAG, AGGTGTTC, AGGTTCCA** |
