# Supplementary material for: Routine Habitat Change: A Source of Unrecognized Transient Alteration of Intestinal Microbiota in Laboratory Mice
Source: PLoS One. 2012 Oct 17;7(10):e47416. doi: 10.1371/journal.pone.0047416 (PMC3474821; doi:10.1371/journal.pone.0047416)
Supplement: Table S3 — NGS taxonomic data of fecal microbiota in mice following a combined facility/cage change. (PDF) [file pone.0047416.s008.pdf]

Table S3. NGS taxonomic data of fecal microbiota in mice following a combined facility/cage change.

	Mouse A						Mouse E					
Taxon	D0	D1	D2	D3	D4	D5	D0	D1	D2	D3	D4	D5
Bacteroidetes;Other;Other;Other;Other	0.2141521	0.2769406	0.2879151	0.2144314	0.3717343	0.2839418	0.1642504	0.2302885	0.2482137	0.2478485	0.2575067	0.2186794
Bacteroidetes;Bacteroidia;Bacteroidales;Other;Other	0.1788466	0.207848	0.2348482	0.1116012	0.2005474	0.1633044	0.1896833	0.1826349	0.2200897	0.223591	0.1541019	0.1606723
Bacteroidetes;Bacteroidia;Bacteroidales;Porphyromonadacea	0.1249159	0.1614442	0.146742	0.0887047	0.1255495	0.0989487	0.1568221	0.1746275	0.235154	0.187943	0.1652547	0.1504819
Firmicutes;Clostridia;Clostridiales;Lachnospiraceae;Other	0.1308001	0.0780428	0.0658916	0.1395461	0.0827113	0.0978122	0.1218899	0.1625467	0.120316	0.1282484	0.1097855	0.0960944
Other;Other;Other;Other;Other	0.0834767	0.0885321	0.0766229	0.1110148	0.0632413	0.0862812	0.0725982	0.0978461	0.0852453	0.0744389	0.0910992	0.1318071
Firmicutes;Clostridia;Clostridiales;Other;Other	0.09345	0.0776437	0.0814804	0.177588	0.0628473	0.1024056	0.0353464	0.0515875	0.0314782	0.0321887	0.0363807	0.0302946
Firmicutes;Bacilli;Lactobacillales;Lactobacillaceae;Lactobacill	0.036627	0.0009121	0.0004268	0.0007904	0.017044	0.0662026	0.1934388	0.0100999	0.0068871	0.0472494	0.1238874	0.1421589
Firmicutes;Clostridia;Clostridiales;Ruminococcaceae;Other	0.0660483	0.0364466	0.0260355	0.0938552	0.0321597	0.0522091	0.0257366	0.0417387	0.0163941	0.0225278	0.0204021	0.0131185
Firmicutes;Other;Other;Other;Other	0.0393697	0.0387838	0.0437381	0.0219276	0.0197603	0.0215229	0.0168448	0.021818	0.0170491	0.0181615	0.025067	0.0377415
Firmicutes;Clostridia;Other;Other;Other	0.0222654	0.0185463	0.0148165	0.0304437	0.0121299	0.0163375	0.0084224	0.0109648	0.0070062	0.007889	0.0068097	0.0064785
Verrucomicrobia;Verrucomicrobiae;Verrucomicrobiales;Verru	0.002618	0.008399	0.014349	0.0015298	0.0025297	0.0021783	0.005799	0.0077005	0.0065299	0.0039867	0.0011796	0.0033891
Firmicutes;Clostridia;Clostridiales;Lachnospiraceae;Paraspor	0.0017204	0.0016342	0.002195	0.0023202	0.0014722	0.0017521	0.0006627	0.0015066	0.0006947	0.0005695	0.0005362	0.0005533
Firmicutes;Bacilli;Lactobacillales;Other;Other	0.0007979	7.60E-05	6.10E-05	0.000102	0.0002488	0.0016811	0.0027614	0.0003627	0.0002382	0.0005906	0.0023056	0.0026975
Firmicutes;Bacilli;Lactobacillales;Lactobacillaceae;Other	0.0006981	0.0005891	0.0006097	0.0004844	0.0004562	0.0004736	0.000718	0.0008928	0.0005359	0.0006117	0.0008043	0.0005764
Proteobacteria;Gammaproteobacteria;Enterobacteriales;Ente	0.0005735	0.0006081	0.0004878	0.0006884	0.0007465	0.000663	0.0005523	0.0005022	0.0005557	0.0007172	0.0005362	0.0005764
Firmicutes;Clostridia;Clostridiales;Lachnospiraceae;Dorea	0.0004737	0.000323	0.0003455	0.0006374	0.0007672	0.0007814	0.0003314	0.0002511	0.0001786	0.0002742	0	2.31E-05
Proteobacteria;Betaproteobacteria;Burkholderiales;Comamor	0.0001995	0.000266	0.0001829	0.0002805	0.0005806	0.0001894	0.0003866	0.0008649	0.0003374	0.0001477	0.0002413	0.0002997
Proteobacteria;Gammaproteobacteria;Pseudomonadales;Pse	0.0002992	0.000304	0.0003658	0.000204	0.0005391	0.0002605	0.0005247	0.0003348	0.0002183	0.0002531	0.0003485	0.0002306
Cyanobacteria;Cyanobacteria;Chloroplast;Streptophyta;Othe	0.0001247	7.60E-05	0.0002236	0.0006629	0.001016	0.0004025	0.0001933	0.0003627	9.92E-05	0.0001477	0.0003485	0.0001383
Firmicutes;Bacilli;Other;Other;Other	0.0002743	3.80E-05	2.03E-05	2.55E-05	0.0001244	0.0003315	0.000718	0.0001116	3.97E-05	0.0003164	0.0005362	0.000853
Bacteroidetes;Bacteroidia;Bacteroidales;Prevotellaceae;Prev	0.0002743	0.000171	0.0002845	0.000255	0.000394	0.0003078	0.0001933	0.000279	0.0002977	0.0002109	0.0002949	0.0003458
Proteobacteria;Gammaproteobacteria;Aeromonadales;Succin	0.0001745	0.000152	0.0001626	0.0003825	6.22E-05	0.0001421	0.0001933	0.0001674	7.94E-05	0.0001055	0.0001877	0.0001844
Actinobacteria;Actinobacteria;Actinomycetales;Micrococcacea	0.0001247	0.000152	0.0001829	0.000102	0.0001451	7.10E-05	8.28E-05	5.58E-05	0.0001786	0.0001055	0.0002145	0.0001614
Firmicutes;Bacilli;Bacillales;Other;Other	0.0001247	0.000171	4.06E-05	0.000153	0.0002488	0.0001657	0.0001657	0.0001116	0.0001191	2.11E-05	2.68E-05	0.0001844
Firmicutes;Bacilli;Lactobacillales;Streptococcaceae;Lactococ	0.0002244	0.000209	0.0001016	0.000102	6.22E-05	7.10E-05	8.28E-05	0.0001674	7.94E-05	0.0001266	0.000134	0.0001614
Proteobacteria;Gammaproteobacteria;Aeromonadales;Succin	0.0001247	9.50E-05	0.0001219	0.0001785	0.0001659	0.0001184	0.0001933	5.58E-05	5.95E-05	0.0001055	5.36E-05	0.0001383
Bacteroidetes;Bacteroidia;Bacteroidales;Bacteroidaceae;Bact	7.48E-05	0.000133	0.0001423	0.000102	0.0001244	0.0001421	8.28E-05	5.58E-05	0.0001985	8.44E-05	0.000134	0.0001153
Actinobacteria;Actinobacteria;Actinomycetales;Nocardiaceae	4.99E-05	0.000133	8.13E-05	0.0001785	0.0001244	2.37E-05	5.52E-05	5.58E-05	0.0002382	6.33E-05	0.000134	9.22E-05
Bacteroidetes;Bacteroidia;Bacteroidales;Porphyromonadacea	4.99E-05	7.60E-05	4.06E-05	0.000102	0.0001244	7.10E-05	2.76E-05	0.0001674	9.92E-05	0.0001055	0.000134	9.22E-05
Actinobacteria;Actinobacteria;Coriobacteriales;Coriobacteria	0	0	4.06E-05	0	0.0001451	2.37E-05	0.0001381	0	7.94E-05	0.0001687	0.000134	0.0002306
Unclassified;Other;Other;Other;Other;Other	0	1.90E-05	2.03E-05	0.0001275	0.0002903	0.0001657	0.0001105	0	5.95E-05	2.11E-05	5.36E-05	0
Proteobacteria;Gammaproteobacteria;Enterobacteriales;Ente	2.49E-05	7.60E-05	8.13E-05	0.000102	0.0001244	9.47E-05	2.76E-05	2.79E-05	0.0001191	8.44E-05	8.04E-05	2.31E-05
Firmicutes;Bacilli;Lactobacillales;Enterococcaceae;Enterococ	4.99E-05	5.70E-05	6.10E-05	5.10E-05	0.0001037	4.74E-05	2.76E-05	0.0001395	3.97E-05	6.33E-05	8.04E-05	0.0001383
Actinobacteria;Actinobacteria;Actinomycetales;Micrococcacea	0.0001496	1.90E-05	8.13E-05	0.000153	6.22E-05	0	8.28E-05	8.37E-05	5.95E-05	0	8.04E-05	6.92E-05
Firmicutes;Clostridia;Clostridiales;Clostridiaceae;Other	4.99E-05	0.000133	8.13E-05	5.10E-05	6.22E-05	2.37E-05	2.76E-05	8.37E-05	1.98E-05	6.33E-05	0.000134	4.61E-05
Proteobacteria;Gammaproteobacteria;Pseudomonadales;Mor	7.48E-05	3.80E-05	4.06E-05	2.55E-05	2.07E-05	0	5.52E-05	2.79E-05	0.0001191	8.44E-05	0.0002145	2.31E-05
Proteobacteria;Alphaproteobacteria;Rhizobiales;Other;Other	4.99E-05	5.70E-05	8.13E-05	7.65E-05	2.07E-05	9.47E-05	0	8.37E-05	5.95E-05	4.22E-05	0	4.61E-05
Proteobacteria;Gammaproteobacteria;Xanthomonadales;Xan	9.97E-05	3.80E-05	4.06E-05	7.65E-05	4.15E-05	0	5.52E-05	2.79E-05	3.97E-05	2.11E-05	5.36E-05	0.0001153
Proteobacteria;Gammaproteobacteria;Enterobacteriales;Ente	4.99E-05	0	4.06E-05	0	0.0001037	0.0001184	0	0.0002511	0	0	0	0
Firmicutes;Clostridia;Clostridiales;Clostridiaceae;Clostridium	9.97E-05	5.70E-05	4.06E-05	7.65E-05	2.07E-05	2.37E-05	2.76E-05	8.37E-05	7.94E-05	0	2.68E-05	2.31E-05
Bacteroidetes;Bacteroidia;Bacteroidales;Prevotellaceae;Othe	2.49E-05	9.50E-05	8.13E-05	2.55E-05	4.15E-05	7.10E-05	5.52E-05	0	5.95E-05	0	2.68E-05	4.61E-05
Firmicutes;Bacilli;Lactobacillales;Leuconostocaceae;Leuconos	4.99E-05	3.80E-05	0	5.10E-05	4.15E-05	4.74E-05	5.52E-05	0.0001116	1.98E-05	4.22E-05	2.68E-05	0
Firmicutes;Bacilli;Lactobacillales;Streptococcaceae;Streptoco	2.49E-05	1.90E-05	4.06E-05	7.65E-05	2.07E-05	2.37E-05	5.52E-05	8.37E-05	1.98E-05	2.11E-05	2.68E-05	6.92E-05
Firmicutes;Bacilli;Bacillales;Bacillaceae;Other	2.49E-05	3.80E-05	0	2.55E-05	8.29E-05	0	0	8.37E-05	7.94E-05	2.11E-05	8.04E-05	2.31E-05
Actinobacteria;Actinobacteria;Coriobacteriales;Coriobacteria	0	0	2.03E-05	0	8.29E-05	0	0	8.37E-05	0	0.0001055	2.68E-05	0.0001153
Firmicutes;Clostridia;Clostridiales;Veillonellaceae;Dialister	0	1.90E-05	2.03E-05	0.000102	4.15E-05	0	2.76E-05	5.58E-05	7.94E-05	0	5.36E-05	2.31E-05
Proteobacteria;Betaproteobacteria;Burkholderiales;Comamor	0	7.60E-05	4.06E-05	2.55E-05	4.15E-05	2.37E-05	2.76E-05	5.58E-05	3.97E-05	4.22E-05	0	4.61E-05
Proteobacteria;Alphaproteobacteria;Rhizobiales;Phyllobacteri	0	1.90E-05	2.03E-05	2.55E-05	8.29E-05	2.37E-05	2.76E-05	2.79E-05	3.97E-05	8.44E-05	5.36E-05	0
Proteobacteria;Epsilonproteobacteria;Campylobacterales;Car	4.99E-05	0	6.10E-05	5.10E-05	2.07E-05	2.37E-05	0	2.79E-05	1.98E-05	6.33E-05	2.68E-05	2.31E-05
Firmicutes;Clostridia;Clostridiales;Lachnospiraceae;Roseburi	0	3.80E-05	2.03E-05	0	4.15E-05	4.74E-05	0.0001381	0	1.98E-05	0	2.68E-05	2.31E-05
Firmicutes;Clostridia;Clostridiales;Veillonellaceae;Megasphae	2.49E-05	1.90E-05	2.03E-05	2.55E-05	6.22E-05	0	2.76E-05	0	5.95E-05	4.22E-05	2.68E-05	4.61E-05
Firmicutes;Erysipelotrichi;Erysipelotrichales;Erysipelotrichace	2.49E-05	7.60E-05	0.0001626	2.55E-05	4.15E-05	2.37E-05	0	0	0	0	0	0
Firmicutes;Clostridia;Clostridiales;Ruminococcaceae;Subdoli	2.49E-05	1.90E-05	0	2.55E-05	4.15E-05	0	5.52E-05	2.79E-05	1.98E-05	4.22E-05	0	9.22E-05
Bacteroidetes;Bacteroidia;Bacteroidales;Rikenellaceae;Alistip	4.99E-05	7.60E-05	4.06E-05	2.55E-05	2.07E-05	0	8.28E-05	0	1.98E-05	0	0	2.31E-05
Firmicutes;Bacilli;Lactobacillales;Leuconostocaceae;Weissella	2.49E-05	1.90E-05	2.03E-05	2.55E-05	2.07E-05	0	2.76E-05	0	1.98E-05	6.33E-05	8.04E-05	2.31E-05
Bacteroidetes;Bacteroidia;Bacteroidales;Porphyromonadacea	0	5.70E-05	4.06E-05	2.55E-05	4.15E-05	0	0	5.58E-05	0	2.11E-05	0	4.61E-05
Proteobacteria;Alphaproteobacteria;Caulobacterales;Cauloba	2.49E-05	9.50E-05	2.03E-05	0	2.07E-05	0	0	0	3.97E-05	2.11E-05	2.68E-05	2.31E-05
Bacteroidetes;Sphingobacteria;Sphingobacteriales;Sphingoba	0	0	2.03E-05	0	4.15E-05	0	0	8.37E-05	1.98E-05	6.33E-05	2.68E-05	0
Proteobacteria;Gammaproteobacteria;Xanthomonadales;Xan	0	0	0	2.55E-05	2.07E-05	9.47E-05	2.76E-05	5.58E-05	0	0	0	2.31E-05
Proteobacteria;Gammaproteobacteria;Pseudomonadales;Mor	0	0	2.03E-05	0	6.22E-05	4.74E-05	0	2.79E-05	3.97E-05	4.22E-05	0	0
Proteobacteria;Gammaproteobacteria;Oceanospirillales;Halo	0	1.90E-05	0	0	2.07E-05	2.37E-05	0	2.79E-05	5.95E-05	0	2.68E-05	4.61E-05
Firmicutes;Bacilli;Bacillales;Planococcaceae;Other	0	0	0	2.55E-05	6.22E-05	2.37E-05	0	5.58E-05	0	2.11E-05	0	0
Firmicutes;Clostridia;Clostridiales;Veillonellaceae;Anaerovibr	0	0	2.03E-05	0	4.15E-05	2.37E-05	0	0	0	6.33E-05	2.68E-05	0
Proteobacteria;Alphaproteobacteria;Rhodospirillales;Acetoba	0	0	4.06E-05	5.10E-05	4.15E-05	0	0	0	1.98E-05	2.11E-05	0	0
Actinobacteria;Actinobacteria;Actinomycetales;Microbacteria	0	0	0	2.55E-05	4.15E-05	2.37E-05	2.76E-05	0	0	0	2.68E-05	2.31E-05
Firmicutes;Bacilli;Lactobacillales;Leuconostocaceae;Oenococ	0	0	2.03E-05	0.000102	4.15E-05	0	0	0	0	0	0	0
Firmicutes;Bacilli;Bacillales;Bacillaceae;Oceanobacillus	0	0	6.10E-05	0	0	0	0	0	0	0	2.68E-05	6.92E-05
Proteobacteria;Betaproteobacteria;Burkholderiales;Alcaligena	0	0	0	0	4.15E-05	0	0	5.58E-05	0	0	0	4.61E-05
Actinobacteria;Actinobacteria;Bifidobacteriales;Bifidobacteria	0	0	0	0	6.22E-05	2.37E-05	2.76E-05	2.79E-05	0	0	0	0
Proteobacteria;Gammaproteobacteria;Other;Other;Other	0	3.80E-05	0	0	0	2.37E-05	0	0	0	0	2.68E-05	4.61E-05
Firmicutes;Clostridia;Clostridiales;Veillonellaceae;Mitsuokella	2.49E-05	0	2.03E-05	0	4.15E-05	2.37E-05	0	0	1.98E-05	0	0	0
Proteobacteria;Betaproteobacteria;Burkholderiales;Other;Ot	2.49E-05	0	0	0	2.07E-05	0	2.76E-05	5.58E-05	0	0	0	0
Proteobacteria;Other;Other;Other;Other	0	0	0	5.10E-05	0	0	0	2.79E-05	1.98E-05	0	0	2.31E-05
Firmicutes;Clostridia;Clostridiales;Veillonellaceae;Acidamino	0	0	2.03E-05	5.10E-05	0	0	0	0	0	2.11E-05	0	2.31E-05
Proteobacteria;Alphaproteobacteria;Sphingomonadales;Sphi	0	0	0	0	6.22E-05	2.37E-05	0	0	0	0	2.68E-05	0
Bacteroidetes;Flavobacteria;Flavobacteriales;Flavobacteriace	0	0	4.06E-05	0	0	0	0	0	0	2.11E-05	2.68E-05	2.31E-05
